# Study on the association between malnutrition, early childhood caries and caries activity among children aged 3–5 years

**DOI:** 10.1186/s12903-024-04802-9

**Published:** 2024-09-03

**Authors:** Duorui Wang, Xinfeng Wang, Caiyun Zhao, Siting Ma, Yanning Zhang, Hong Shi

**Affiliations:** 1 Department of Pediatric Dentistry, Hospital of Stomatology and Hebei Provincial Key Laboratory of Stomatology, Hebei Medical University, Shijiazhuang, China; 2 Qingdao Stomatological Hospital, Qingdao University, No.17, Daode County Road, Zhongshan Road Street, Shinan District, Qingdao City, China; 3 Department of oral pathology, Hospital of Stomatology and Hebei Provincial Key Laboratory of Stomatology, Hebei Medical University, Shijiazhuang, China

**Keywords:** Early childhood caries, Caries activity, Malnutrition; obesity, Underweight

## Abstract

**Background:**

This study aims to investigate the association between malnutrition and early childhood caries (ECC) and caries activity among children aged 3–5 years, in order to provide a theoretical basis for preventing and blocking ECC and improving malnutrition.

**Methods:**

Children aged 3–5 years from six kindergartens in Zhao Xian, China were enrolled in this study. The decayed, missing, filled teeth (dmft) of all children were examined and recorded. The Cariostat method was used to detect dental caries activity, collect anthropometric data and measure haemoglobin concentration. Parents were asked to complete a questionnaire on the general characteristics and oral health behaviour of the participants. The “Growth Standards for Chinese Children Under 7 Years Old” was used to assess the nutritional status of all participating children. Wilcoxon rank sum test and multivariate logistic regression analysis were used to analyse and evaluate the relationship between ECC, caries activity and malnutrition.

**Results:**

A total of 635 children who met the criteria were included in this study. After adjusting for confounding factors, logistic regression showed that the risk of ECC was significantly increased in underweight children compared with normal children (OR = 5.43, *P* < 0. 05); compared with normal children, the risk of ECC decreased in overweight and obese children (OR = 0.31, *P* < 0.001); underweight children had higher caries severity than normal weight children, and the difference was statistically significant (OR = 2.69, *P* < 0. 05); stunted children had higher caries severity than normal weight children and the difference was statistically significant (OR = 2.28, *P* < 0.05); underweight was positively associated with caries activity and the association was statistically significant (OR = 2.33, *P* < 0. 05); stunting was positively associated with caries activity and the association was statistically significant (OR = 2.1, *P* < 0.05); overweight and obesity were negatively associated with caries activity and the association was statistically significant (OR = 0.61, *P* < 0.05).

**Conclusions:**

The risk of ECC among children aged 3–5 years was positively associated with undernutrition and negatively associated with overnutrition. The severity of ECC among children aged 3–5 years was positively associated with undernutrition. The caries activity among children aged 3–5 years was positively associated with undernutrition and negatively associated with overnutrition.

**Supplementary Information:**

The online version contains supplementary material available at 10.1186/s12903-024-04802-9.

## Background

Early childhood caries (ECC), formerly referred to as nursing bottle caries and baby bottle tooth decay remains a significant chronic disease of childhood and public health problems [1]. The American Academy of Pediatric Dentistry (AAPD) defines ECC as the presence of one or more decayed (non-cavitated or cavitated), missing (as a result of caries), or filled tooth surfaces in any primary tooth in a child 71 months of age or younger [2]. According to a report by the International Association of Paediatric Dentistry (IAPD), which summarised the results of 72 studies published between 1998 and 2018, the prevalence of ECC in children aged 3, 4 and 5 years was 43%, 55% and 63%, respectively [3]. And the Fourth China Oral Health Epidemiological Survey showed that in 2018, the prevalence of caries in 3, 4, and 5 year old children in China was 50.8%, 63.6%, and 71.9%, respectively, but the treatment rates were only 1.5%, 2.9%, and 4.1%, respectively [4], and about 40% of the cases would recur within one year even after treatment [5].

ECC is a multifactorial disease whose onset and development are associated not only with poor dietary habits, family economic status and oral hygiene practices, but also with childhood malnutrition [2, 6]. Malnutrition is an imbalance between the body’s nutritional requirements and intake and includes undernutrition (wasting, stunting, low body weight), overnutrition (overweight, obesity) and micronutrient malnutrition [7]. Malnutrition reduces the production and quality of saliva in children, which in turn affects the ability of the mouth to clean and cushion the teeth, increasing the risk of exposure to cariogenic bacteria and ultimately contributing to the development and progression of ECC [8, 9]. ECC can cause symptoms such as pain, facial swelling and difficulty chewing, which can affect a child’s intake and absorption of nutrients [10], leading to long-term health problems and malnutrition. In addition, both ECC and malnutrition are influenced by socioeconomic factors such as poverty, education, sanitation, nutrition and medical care, which together increase the risk of ECC and malnutrition in children [11, 12]. Therefore, the possible association and bidirectional relationship between ECC and malnutrition deserves to be studied in depth. 

The conclusions of current studies on the relationship between ECC and malnutrition are inconsistent. For example, Leonor [13] found that children with malnutrition had a higher decayed, missing, and filled tooth surfaces (dmfs) than normal children; Olatosi [14] found that the severity of ECC in wasting children was significantly higher than that in normal and overweight children; and Li [15] studied 233 preschool children in Chengdu and found that the number of caries-loss fillings was negatively associated with Weight-for-height Z-score(WHZ), and that the greater the degree of malnutrition, the greater the degree of caries.

Some studies have found that overweight and obese children have a higher risk and severity of ECC, for example, Manohar [16] concluded that overweight and obese children have a higher risk of dental caries, and Bafti [17] found that the caries rate in primary teeth of children aged 3–6 years decreased with increasing body weight, suggesting that underweight children are more susceptible to caries. On the other hand, Singh, Manriquez [18, 19] found no association between ECC and malnutrition. These findings suggest that the relationship between ECC and malnutrition may be influenced by a variety of factors that need to be investigated in further studies.

In predicting the risk of ECC, the caries activity test (CAT) is one of the most important indicators for screening susceptible children [20]. Studies have shown that caries activity (CA) is closely related to the occurrence and development of ECC, and the use of caries activity to assess the level of caries risk in children is of great importance in guiding dentists and parents of children to carry out the necessary early intervention [21]. In a study by Wang [22], caries activity was found to be associated with both inadequate dietary intake and the degree of dietary imbalance, both of which may cause malnutrition [23, 24], which makes us doubt whether caries activity is related to malnutrition, but no relevant studies have been reported.

This study analysed the association between malnutrition and ECC and caries activity in children aged 3–5 years in order to formulate appropriate caries prevention strategies for children with malnutrition, to take preventive measures and to provide a rational basis for further prevention of ECC.

## Materials and methods

### Study population

In this study, 887 children aged 3–5 years enrolled in six kindergartens in the Zhao Xian area of Shijiazhuang were studied from March 2022 to September 2022 using multi-stage stratified sampling. The specific sampling procedure was as follows: first, six kindergartens in Zhao Xian area of Shijiazhuang were randomly selected, and each kindergarten was stratified according to primary, secondary and tertiary classes, listing all classes in primary, secondary and tertiary classes to ensure that each class had a unique number. A random number generator was then used to randomly select 2 classes from each stratum of small, medium and large classes, and children in the selected classes who met the inclusion criteria were included. In the end, 635 children returned questionnaires and had complete data.

**Inclusion criteria:**

(1) Aged 3–5 years and living with a parent/guardian; (2) At the stage of primary dentition

**Exclusion criteria:**

(1) Children with any psychiatric or systemic disease that could affect the results of the oral health examination or the caries activity test; (2) Use of antibiotics in the month prior to the examination or use of hormones or immunosuppressants within six months; (3) Suffering from digestive, endocrine and other systemic diseases, metabolic diseases, acute and chronic inflammatory diseases

### Sample size estimation

The sample size was estimated according to the sample content formula N = t ^2^ PQ/d^2^ for cross-sectional studies, where N is the sample size, t is the t-score corresponding to the confidence level, P is the overall prevalence, Q = 1-P, and d is the acceptable error. According to the fourth Chinese oral health epidemiological survey in 2018, the prevalence of ECC was 62.5% [25]. The error was set as 0.1 times the prevalence, 62.5% * 0.1 = 6.25%, with a statistical test level of α = 0.05, and *N* = 230 from the formula.Finally, 635 valid samples were included in this study, and samples with invalid or missing data were excluded after screening.

### Ethical review

The study was approved by the Medical Ethics Committee of the Stomatological Hospital of Hebei Medical University (Ethics No. [2018] 028) and was conducted in accordance with the tenets of the Declaration of Helsinki. Legal guardians were informed of the purpose, significance, and health implications of the study and signed an informed consent form before the study began.

### Research tools and methodology

#### Oral examination

The oral examination was conducted by two trained paediatric dentists who passed the kappa consistency test with kappa values of 0.76 and 0.78. Two caries diagnostic criteria were used to make the examination as accurate as possible: the World Health Organization’s caries examination and diagnostic criteria were used to assess caries in pits and fissures, and caries that had become cavities on smooth surfaces [26]; The International Caries Detection and Assessment System (ICDAS-II) is used to detect early caries [27]. Under natural light, the children’s oral condition was examined with sterile stomatoscope and probe, and the dmft was recorded, and if necessary, surfaces were probed using the Community Periodontal Index (CPI) probe to determine the continuity and roughness of the enamel surface. The samples were first divided into caries-free and ECC groups according to dmft, and then the ECC group was divided into three groups according to caries severity, with 1 ≤ dmft ≤ 4 as the low caries group, 5 ≤ dmft ≤ 8 as the medium caries group, and dmft ≥ 9 as the high caries group. This classification was based on previous studies [28, 29].

### Caries activity test

Two trained paediatric dentists performed the caries activity test on the children in the study. Mouth rinsing was required prior to sampling so that large amounts of food debris would not interfere with the test results. The examiners used a dental mirror to gently open the corner of the child’s mouth and wiped the buccal neck of the maxillary molars and the labial neck of the mandibular anterior teeth 3–5 times with a special sterile cotton swab to collect plaque, and then quickly placed the swabs in 2.5 ml Cariostat reagent bottles (Beijing Gunda Medical Technology Co, Ltd.) with numbering; they were placed in the constant temperature incubator of 37℃ for 48 ± 4 h within 4 h, and then one of the examiners placed the reagents under natural light, and the results were read against a standard colour card and grouped. The parents of the children were informed of the results by telephone and given advice on how to improve oral hygiene. The results, i.e. the caries activity index, were scored as 0, 0.5, 1.0, 1.5, 2.0, 2.5 and 3.0, where 0–1.0 was the low caries activity group (L group), i.e. low caries risk, 1.5 was the medium caries activity group (M group), i.e. medium caries risk, and 2.0–3.0 was the high caries activity group (H group), i.e. high caries risk [21].

### Examination of anthropometric data and assessment of malnutrition

Weight and height measurements were accurately recorded by a professionally trained person according to the ‘Anthropometric Methods for Health Surveillance of the Chinese Population’ [30] with an accuracy of 0.1 kg and 0.1 cm, respectively, and the height and weight values were used to calculate the body mass index (BMI): BMI = body weight/height^2^ (kg/m^2^). The nutritional status of the children was assessed using the Chinese Growth Standards for Children Under 7 Years of Age [31](An additional movie file shows this in more detail [see Additional file 1]). The standard deviation was first calculated based on the child’s age, height, weight and BMI against additional file 2, and then the standard deviation was used to determine the child’s nutritional status against additional file 1. The weight-for-age Z-score (WAZ), height-for-age Z-score (HAZ) and BMI-for-age Z-score (BAZ) were calculated according to the height, weight and BMI of the children. WAZ < -2 was considered as underweight and the rest as normal; HAZ < -2 was considered as stunting and the rest as normal; BAZ ≥ 1 was considered as overweight and obesity, -2 ≤ BAZ < 1 was considered as normal and BAZ < -2 was considered as wasting.

### Blood sampling and anaemia diagnosis

A trained community nurse collected 10µL of blood from a fingertip vein and used a haemoglobin analyser to measure the children’s haemoglobin concentration, then used the WHO criteria for the diagnosis of anaemia [32]: haemoglobin concentration less than 110 g/L in children aged 6 months to 5 years, children with haemoglobin concentration less than 110 g/L in children aged 6 months to 5 years were classified as anaemic and the rest as non-anaemic. Anaemia was used to assess whether the children were suffering from micronutrient-related malnutrition.

### Questionnaire survey

The questionnaire was designed with reference to the Fourth Chinese Oral Health Epidemiological Survey Report [25]. The questionnaire included family income, parents’ highest level of education, mode of delivery, whether the baby was born preterm, birth weight, maternal health during pregnancy, feeding mode before 6 months of age, frequency of falling asleep with nipple or bottle, parents chewing food to feed the child, parents sharing a set of utensils with the child, time of snacking, frequency of snacks or sweets, whether snacks were eaten before bedtime, whether snacks were eaten before bedtime or after brushing teeth, frequency of sugary drinks, whether parents supervised brushing, frequency of dental visits, frequency of brushing, frequency of using toothpaste, whether parents supervised brushing, frequency of dental visits, frequency of brushing, use non-fluoride or low-fluoride toothpaste, frequency of eating fruit, cereals, vegetables, yoghurt, plain milk. The questionnaire is completed by the child’s parent or guardian. Once the questionnaire has been completed by the child’s parent/guardian, the parent/guardian will be asked to complete the questionnaire again, and if there are any options that differ from the first time, the questionnaire will be checked by telephone or in a face-to-face interview to verify the accuracy and validity of the questionnaire.

### Statistical analyses

SPSS 27.0 was used for statistical analysis of the data. In descriptive statistics, conformity of measurement data to normality was expressed as mean ± standard deviation, conformity to skewness was expressed as median and interquartile range, and count data were expressed as constitutive ratio (%). As the data distribution did not conform to normality and/or variance, non-parametric tests (Wilcoxon rank sum test and chi-squared test) were used to compare dmft, caries prevalence and caries activity in different nutritional status subgroups, and one-way analyses of general characteristics and oral health behaviours of the children were performed using chi-squared tests to assess their association with ECC. Variables with significant differences in the univariate analyses were included in a multivariate logistic regression model to assess the association between malnutrition and the presence of caries, caries severity and caries activity, with the criterion for inclusion in the regression model being *P* ≤ 0.05.

## Results

### General characteristics and oral health behaviours of the participants

A total of 635 children aged 3–5 years were enrolled between March 2022 and September 2022, of whom 527 had caries and 108 were caries-free. The general characteristics and oral health behaviours of the participants are shown in Table 1(at the end of the document text file). Annual household income, frequency of consumption of sugary drinks, use non-fluoride or low-fluoride toothpaste, frequency of consumption of vegetables and frequency of consumption of yoghurt had a significant association with caries rate (*P* < 0. 05), with the highest caries rates in children with an annual family income of less than 5000 Yuan, who drank sugary drinks more than once a day, use non-fluoride toothpaste, consumed vegetables less than once a day and yoghurt more than twice a day.

**Table 1:** Study participants’ general characteristics and oral health behaviors

Variables		*n*	Prevalence of ECC [*n* (%)]	*P*-value
age				>0.05
	3 years old	130	102 (78.5)	
	4 years old	266	219 (82.3)	
	5 years old	239	206 (86.2)	
gender				>0.05
	male	334	274 (82)	
	women	301	253 (84.1)	
Monthly household income				0.002*
	< 5000 yuan	283	250 (88.3)	
	5,000 yuan − 20,000 yuan	306	237 (77.5)	
	≥ 20,000 yuan	46	40 (87.0)	
Highest educational level of the father				>0.05
	Junior high school and below	247	202 (81.8)	
	High school or econdary school	215	183 (85.1)	
	University and above	173	142 (82.1)	
Mother’s highest level of education				>0.05
	Junior high school and below	226	191 (84.5)	
	High school or secondary school	225	184 (81.8)	
	University and above	184	152 (82.6)	
Mode of delivery				>0.05
	natural childbirth	398	325 (81.6)	
	caesarean section	237	202 (85.2)	
Whether the baby was born prematurely				>0.05
	no	604	503 (83.2)	
	Yes	31	24 (77.4)	
birth weight				>0.05
	< 2.5 kg	22	19 (86.3)	
	2.5–4 kg	555	459 (82.7)	
	> 4 kg	58	49 (84.4)	
Maternal condition during pregnancy				>0.05
	normal	625	520 (83.2)	
	Malnutrition	2	2 (100)	
	Systemic diseases	8	5 (62.5)	
Feeding before 6 months of age is				>0.05
	breast feeding	491	414 (84.3)	
	mixed feeding	108	86 (79.6)	
	artificial feeding	36	27 (75)	
Frequency of night feeds with nipple or bottle to sleep				>0.05
	never	259	207 (79.9)	
	< 1 time/day	302	260 (86)	
	≥ 1 time/day	74	60 (81)	
Age of withdrawal from night feeding				>0.05
	Under 6 months	18	12 (66.6)	
	6–12 months	118	98 (83)	
	1-1.5 years	356	298 (83.7)	
	> 1.5 years	143	119 (83.2)	
Frequency of parents chewing food to feed their children				>0.05
	never	562	469 (83.4)	
	< 1 time/day	70	55 (78.5)	
	≥ 1 time/day	3	3 (100)	
Do parents and children share a set of cutlery				>0.05
	no	485	404 (83.2)	
	yes	150	123 (82)	
Snack time				>0.05
	mealtime	62	48 (77.4)	
	Between meals	573	479 (83.5)	
Frequency of snacks or sweets				>0.05
	never	24	18 (75)	
	1 time/day	213	178 (83.5)	
	≧ 2 times/day	237	203 (85.6)	
	< 1 time/day	161	128 (79.5)	
Frequency of consumption of sugar-sweetened beverages				0.044*
	never	281	238 (84.7)	
	1 time/day	24	19 (90.5)	
	≧ 2 times/day	92	82 (89.1)	
	< 1 time/day	241	188 (78)	
Whether or not to snack before bed				>0.05
	yes	378	317 (83.9)	
	no	257	210 (81.7)	
Frequency of the child’s visits to the dentist				>0.05
	Every 6 months	183	142 (77.6)	
	Every 12 months	44	36 (81.8)	
	When you have a toothache or find a “cavity”	381	326 (85.6)	
	never	27	23 (85.2)	
Children brush their teeth as often as				>0.05
	≥ 2 times/day	137	112 (81.7)	
	1 time/day	394	326 (82.7)	
	< 1 time/day	104	89 (85.5)	
Whether parents help or supervise tooth brushing				>0.05
	never	325	273 (84)	
	<1 time/day	283	234 (82.7)	
	≥ 1 time/day	27	20 (74.1)	
Use non-fluoride or low-fluoride toothpaste?				0.002*
	low-fluoride toothpaste	291	271 (78.8)	
	non-fluoride toothpaste	344	256 (88)	
Frequency of eating fruits and vegetables				0.043*
	≧ 2 times/day	331	263 (79.5)	
	1 time/day	235	203 (86.4)	
	< 1 time/day	69	61 (88.4)	
Frequency of eating cereals				>0.05
	≧ 2 times/day	279	231 (82.7)	
	1 time/day	270	218 (80.7)	
	< 1 time/day	86	78 (90.6)	
Frequency of yoghurt consumption				0.047*
	≧ 2 times/day	132	116 (87.9)	
	1 time/day	165	142 (86.1)	
	< 1 time/day	338	269 (79.6)	
Frequency of pure milk intake				>0.05
	≧ 2 times/day	87	77 (88.5)	
	1 time/day	305	255 (83.6)	
	< 1 time/day	243	195 (80.2)	

*Statistical difference *P* < 0.05

### Malnutrition in children of different ages and sexes

Table 2 shows the malnutrition status of children of different ages and sexes. A total of 334 boys and 301 girls were included in this study, with an overall rate of underweight of 8 per cent, stunting of 9.1 per cent and obesity of 15.2 per cent. A total of 130 children aged 3 years, 266 children aged 4 years and 239 children aged 5 years were included in this study.The overweight and obesity rates of 3, 4 and 5 year old children gradually increased with age (10.8%, 13.5%, 19.7%, *P*<0.05) and the difference was statistically significant.

**Table 2:** Malnutrition status of children of different ages and sexes

variable	*n*	Low body weight rate	Growth retardation rate	Wasting rate	Obesity and overweight rate
		*P*-value	*P*-value	*P*-value	*P*-value
gender		>0.05	>0.05	>0.05	>0.05
man	334	27(8%)	31(9.2%)	17(5%)	49(14.6%)
woman	301	24(7.9%)	27(8.9%)	11(3.6%)	48(15.9%)
age		>0.05	>0.05	>0.05	0.045
3	130	12(9.2%)	9(6.9%)	7(5.4%)	14(10.8%)
4	266	21(7.9%)	23(8.6%)	14(5.3%)	36(13.5%)
5	239	18(7.5%)	26(10.9%)	7(2.9%)	47(19.7%)

### Caries status and caries activity of children in different malnutrition subgroups

Table 3 shows the caries status and caries activity status of children with different malnutrition status. The dmft, caries prevalence and high caries activity in low weight children were higher than that of normal weight children and the difference was statistically significant (*P* < 0.01); Children with stunting had higher dmft and high caries activity rates than children with normal growth and development, and the difference was statistically significant (*P* < 0.05); The differences in dmft, caries rate and high caries activity rate between the normal, wasting and overweight obese groups were statistically significant (*P* < 0.05).

**Table 3:** Caries status and high caries activity rate of children in different nutritional status groups

Variables	dmft	*P*-value	Prevalence of ECC	*P*-value	High caries activity rate	*P*-value
WAZ						
normal	6 (8)	< 0.001*	478 (81.8)	0.009*	289 (49.5)	0.004*
low weight	9 (6)		49 (96.1)		36 (70.6)	
HAZ						
normal	6 (8)	0.02*	474 (82.1)	>0.05	287 (49.7)	0.022*
stunting	8.5 (7)		53 (91.4)		38 (65.5)	
BAZ						
normal	6 (9)	< 0.001*	437 (85.7)	< 0.001*	268 (52.5)	0.011*
wasting	7.5 (7)		27 (96.4)		19 (67.9)	
overweight and obese	4 (8)		63 (64.9)		38 (39.2)	
anaemia						
there are	6 (8)	>0.05	481 (82.6)	>0.05	260 (44.7)	>0.05
not have	8 (8)		46 (86.8)		27 (50.9)	

*Statistical difference *P* < 0.05; dmft is expressed as median (interquartile spacing); ECC prevalence and high caries activity rates are expressed as n( %)

### Multifactorial logisitc regression analysis of ECC prevalence and malnutrition

Multifactorial logistic regression analysis was used, with the dependent variable being whether or not the person had ECC. *P* ≤ 0.05 for household income, sugary drinks, fluoride in toothpaste, frequency of vegetables and frequency of yoghurt after univariate analysis by Table 1, which included the above factors in the regression model of the variables. In this case, model 1 is without any confounding factors and model 2 includes the above five confounding factors. The statistical results are presented in Table 4. In model 2, corrected for confounders, showed that low weight children had a significantly increased risk of ECC compared to normal weight children (OR = 5.43, 95% CI = 1.27-23; *P* < 0.05); overweight-obese children had a significantly decreased risk of ECC compared to normal weight children (OR = 0.31, 95% CI = 0.18–0.52; *P* < 0.001).

**Table 4:** Multivariate logistic regression analysis of ECC Prevalence and malnutrition

Variables	OR	95% CI	*P*-value
WAZ			
Model 1	5.43	1.30 ~ 22.69	0.02*
Model 2	5.43	1.27–23.08	0.02*
HAZ			
Model 1	2.30	0.89 ~ 5.90	>0.05
Model 2	2.53	0.96 ~ 6.65	>0.05
BAZ^1^			
Model 1	4.51	0.60 ~ 33.70	>0.05
Model 2	4.80	0.62 ~ 37.06	>0.05
BAZ^2^			
Model 1	0.31	0.19 ~ 0.05	< 0.001*
Model 2	0.31	0.18 ~ 0.52	< 0.001*
Anaemia			
Model 1	1.38	0.60 ~ 3.14	>0.05
Model 2	1.37	0.58 ~ 3.21	>0.05

*Statistical difference *P* < 0.05; WAZ for low body mass group compared to normal group; HAZ for growth retardation group compared to normal growth and development group; BAZ^1^ for wasting group compared to normal group; BAZ^2^ for overweight and obese group compared to normal group

### Multiple ordered logistic regression analysis of ECC severity and malnutrition

Multiple ordered logistic regression analyses were used, with the dependent variable being the group divided into low, medium and high caries groups according to caries severity. In this case, model 1 was without confounders and model 2 included the five confounders with *P* ≤ 0.05 in Table 1. All of the above models meet the assumption of parallelism in multivariate ordered logistic regression. The statistical results, as shown in Table 5, showed that in model 2, corrected for confounders, showed that low weight children had higher caries severity compared to normal weight children, and the difference was statistically significant (OR = 2.69, 95% CI = 0.39 ~ 1.58; *P* < 0. 05); and children with stunting had higher caries severity compared to normal children and the difference was statistically significant (OR = 2.28, 95% CI = 0.26 to 1.38; *P* < 0.05).

**Table 5:** Multivariate logistic regression analysis of ECC severity and malnutrition

Variables	OR	95% CI	*P*-value
WAZ			
Model 1	2.46	0.31 ~ 1.48	0.002*
Model 2	2.69	0.39 ~ 1.58	0.001*
HAZ			
Model 1	2.01	0.15 ~ 1.25	0.012*
Model 2	2.28	0.26 ~ 1.38	0.004*
BAZ^1^			
Model 1	1.09	-0.62 ~ 0.81	>0.05
Model 2	1.23	-0.52 ~ 0.94	>0.05
BAZ^2^			
Model 1	0.85	-0.63 ~ 0.33	>0.05
Model 2	0.81	-0.69 ~ 0.29	>0.05
Anaemia			
Model 1	1.30	-0.29 ~ 0.83	>0.05
Model 2	1.22	-0.37 ~ 0.77	>0.05

*Statistical difference *P* < 0.05; WAZ for low body mass group compared to normal group; HAZ for growth retardation group compared to normal growth and development group; BAZ^1^ for wasting group compared to normal group; BAZ^2^ for overweight and obese group compared to normal group

### Multiple ordered logistic regression analysis of caries activity and malnutrition

Multiple ordered logistic regression analyses were used with the dependent variable being the classification into low, medium and high caries activity groups based on the caries activity test scores. In this case, model 1 had no confounders and model 2 included five confounders with *P* ≤ 0.05 in Table 1. All the above models met the assumption of parallelism in multivariate ordered logistic regression. The statistical results are shown in Table 6, where Model 2, corrected for confounders, showed that the effect of low body weight on caries activity was statistically significant (OR = 2.33, 95% CI = 0.22 ~ 1.47; *P* < 0.05); the effect of stunting on caries activity was statistically significant (OR = 2.1, 95% CI = 0.17 ~ 1.31; *P* < 0. 05); and the effect of overweight and obesity on caries activity was statistically significant (OR = 0.1 ~ 0.05). was statistically significant (OR = 0.61, 95% CI = -0.89 ~ 0.05; *P* < 0.05).

**Table 6:** Multivariate logistic regression analysis of caries activity and malnutrition

Variables	OR	95% CI	*P*-value
WAZ			
Model 1	2.33	0.23 ~ 1.45	0.007*
Model 2	2.33	0.22 ~ 1.47	0.007*
HAZ			
Model 1	1.94	0.10 ~ 1.22	0.02*
Model 2	2.10	0.17 ~ 1.31	0.011*
BAZ^1^			
Model 1	1.76	-0.22 ~ 1.35	>0.05
Model 2	1.76	-0.23 ~ 1.37	>0.05
BAZ^2^			
Model 1	0.61	-0.90~-0.08	0.019*
Model 2	0.62	-0.89~-0.05	0.027*
Anaemia			
Model 1	1.24	-3.32 ~ 0.77	>0.05
Model 2	1.18	-0.39 ~ 0.73	>0.05

*Statistical difference *P* < 0.05; WAZ for low body weight group compared to normal group; HAZ for stunting group compared to normal group; BAZ^1^ for wasting group compared to normal group; BAZ^2^ for overweight and obese group compared to normal group

## Discussion

The main findings of this study were: low weight children had an increased risk of ECC, higher severity of ECC and higher caries activity compared to normal children; children with stunting had a higher severity of ECC and higher caries activity compared to normal children; and overweight and obese children had a reduced risk of ECC and lower caries activity compared to normal children.

Our study showed that malnutrition was significantly associated with ECC, where caries was more severe in children with low body weight and stunting, and the risk of ECC was higher in children with low body weight, which was consistent with the findings of Leonor [13] and Olatosi [14]. Shen [33], in a 10-month longitudinal study of 772 preschool children in Liaoning Province, found that there was a negative correlation between dmft and HAZ and WAZ, and that children with low body weight were more prone to caries, which is also consistent with our findings. These findings may be due to the fact that the exocrine gland system may be impaired when children become malnourished [8], and salivary gland atrophy reduces the oral defences against infection and their ability to buffer plaque acids [8], thereby increasing the risk of caries [9]. However, Sheller [34] found no significant association between different weight subgroups and ECC after a retrospective case study, the reason for the discrepancy in the results may be due to the fact that the sample population of the study was younger children undergoing dental treatment under general anaesthesia, which may result in some group heterogeneity, and secondly, the difference in sample size and the confounders used for adjustment (e.g. gender, age, family income, oral hygiene practices, etc.) variations may also lead to different results. In addition, a longitudinal study by Eva [35] showed that the risk of stunting in children with severe caries was about twice that of normal children. This may be due to the restricted dietary choices of caries-affected children, resulting in deficiencies of essential nutrients [36]. Therefore, the role of undernutrition and ECC may be reciprocal and there are many biological, behavioural and environmental factors that may mediate or modulate this relationship. Studies have shown that malnutrition has multiple effects on oral tissues and the subsequent development of oral disease, which in turn accelerates the progression of oral disease by altering tissue homeostasis, decreasing resistance to plaque biofilms and reducing the capacity for tissue repair [37]. Children with malnutrition have poorer overall health, development and quality of life, and a higher risk of dental caries [38].Left untreated, ECC can lead to malnutrition by causing pain or tooth loss and interfering with eating [39, 40]. And the pain associated with dental caries can also lead to sleep deprivation and glucocorticoid and growth factor regulation, which can affect metabolism and nutrition [41]. In addition, malnutrition and ECC are also the result of social justice issues [11], both of which are more prevalent in lower socioeconomic classes and in countries with economies in transition [12].

Our study showed that overnutrition was significantly associated with ECC and that overweight obese children were less likely to develop caries and had relatively less caries severity compared to normal children.Bafti [17] found that caries in the deciduous teeth of 3–6 year old children decreased with increasing weight. Kumar [42] found that in families with high socio-economic status, the prevalence of dental caries in overweight children was approximately 71% lower than in normal weight children. These findings may be due to the increased oral resistance of overnourished children, which contributes to the removal and inhibition of cariogenic bacteria [43]. Studies have shown that overweight and obese children in China are more likely to come from families with relatively higher incomes, and families with relatively higher incomes pay more attention to their children’s oral health [44], resulting in overnourished children being less likely to develop dental caries. However, Paisi [45] found no association between obesity and dental caries in a cross-sectional study of 1250 preschool children. the reason for the discrepancy may be due to the different diagnostic criteria, which used BMI and waist circumference to diagnose obesity, whereas the present study used diagnostic criteria based on the calculation of body measurements, It may also be due to the difference in the target populations, which were developed countries and preschool children, whereas this study used diagnostic criteria calculated on the basis of body measurements, and to the difference in the target population, which were developed countries and preschool children. The sample population for this study came from a different region to the sample population for this study, so differences in diet, culture and economy may also have influenced the results. In addition, Yao [46] found that obesity was associated with high caries prevalence in a cross-sectional study of children aged 5–12 years in southern Anhui, China, suggesting that overnutrition may have different effects on caries in preschool and school-aged children, and that the relationship between caries and overnutrition in children of different ages needs further investigation.

Our study showed that caries activity was positively associated with undernutrition and negatively associated with overnutrition. Zhang [47] studied 329 children aged 3–5 years in Hohhot and found that the higher the caries activity, the lower the BMI in different BMI subgroups, which is basically consistent with our findings. The negative association between caries activity and overnutrition may be due to (1) Dietary structure and frequency: Chinese children eat only a few small snacks between meals in addition to a well-structured main meal each day [48]. In our study, around 63 per cent of children never ate a snack or ate a snack once a day or less.This pattern may reduce the frequency of carbohydrate intake, which is a risk factor for caries development because frequent intake of fermentable carbohydrates leads to more acid erosion of the enamel. (2) Parental influence and socio-economic factors: higher socio-economic status tends to be associated with over-nutrition in China, and may be associated with easier access to dental care and oral hygiene products [44].  

In addition, the present study did not find any relationship between micronutrient-related malnutrition and ECC and caries activity, which may be due to the fact that anaemia was used as a single indicator to diagnose micronutrient-related malnutrition in the present study, and even though one of the most common clinical manifestations of micronutrient-related malnutrition is anaemia [49], there are still some micronutrient-related malnutrition that do not present with anaemia. Therefore, the relationship between this type of malnutrition and ECC needs to be further explored in the future.

In this study, the overweight prevalence of children aged 3–5 years gradually increased with age, which is consistent with the report by Hu [50], suggesting that we need a series of parental interventions to prevent over-nutrition with increasing age. In addition, the prevalence of caries in children aged 3–5 years in our study gradually increased with age, which may be due to the increased exposure of primary teeth to the oral cavity and the lack of good oral hygiene and dietary habits in younger children, as well as the lack of parental supervision. Interestingly, in our study we found that the prevalence of caries was higher in boys than in girls, although the difference was not statistically significant, which is contrary to our common sense and may be due to the small sample size. Our study found lower caries rates in children using low-fluoride toothpaste, and previous studies have shown the importance of using fluoridated toothpaste for caries prevention in younger children [51], where fluoride can delay demineralisation and promote enamel remineralisation.

Our study has a number of limitations. Firstly, this was a cross-sectional study with inherent design limitations, such as the possibility of selection bias, the inability to determine temporal relationships or correlations of findings over time, and the inability to draw conclusions about causality. Secondly, as with any cross-sectional survey, there may be recall bias in the completion of questionnaires by the children’s parents or guardians. Therefore, the generalisability of this study may be limited and should be interpreted with caution. In the future, based on the results of this analysis, our team will conduct a longitudinal study to determine the impact of different types of malnutrition on ECC and caries activity in order to improve the oral health and general health of young children.

## Conclusion

The risk of ECC in children aged 3–5 years was positively associated with undernutrition and negatively associated with overnutrition; the severity of ECC in children aged 3–5 years was positively associated with undernutrition; and caries activity in children aged 3–5 years was positively associated with undernutrition and negatively associated with overnutrition.

### Electronic supplementary material

Below is the link to the electronic supplementary material.

Supplementary Material 1: Growth Standards for Chinese Children Under 7 Years Old (Additional file 1).

Supplementary Material 2: Evaluation table for standard deviation (Additional file 2).

## Data Availability

The datasets used and analyzed during the current study are available from the corresponding author on reasonable request.

## Abbreviations

dmft: Dacayed, missing, filled tooth
ECC: Early childhood caries
AAPD: American academy of pediatric dentistry
IAPD: International association of pediatric dentistry
CAT: Caries activity test
CA: Caries activity
WHO: World health organization
ICDAS: International caries detection and assessment system
CPI: Community periodontal index
BMI: Body mass index
WAZ: Weight-for-age Z-score
HAZ: Height-for-age Z-score
BAZ: BMI-for-age Z-score

## References

[1] Zhang Y, Wang X, Liu X, Li J. Title of the article. J Name, 45(3), 123–34.

[2] American Academy of Pediatric Dentistry. Policy on early childhood caries (ECC): unique challenges and treatment options. Pediatr Dent. 2008;30:44–6. 19216382

[3] Tinanoff N, Baez RJ, Diaz GC, et al. Early childhood caries epidemiology, aetiology, risk assessment, societal burden, management, education, and policy: global perspective. Int J Paediatr Dent. 2019;29(3):238–48. 31099128 10.1111/ipd.12484

[4] Wang X. Report of the fourth national oral health epidemiological survey. Beijing: People’s Health Publishing House; 2018.

[5] Berkowitz RJ, Amante A, Kopycka-Kedzierawski DT, et al. Dental Caries Recurrence following clinical treatment for severe early childhood caries. Pediatr Dent. 2011;33(7):510–4. 22353412

[6] Tsang C, Sokal-Gutierrez K, Patel P, et al. Early Childhood Oral Health and Nutrition in Urban and Rural Nepal. Int J Environ Res Public Health. 2019;16(14):2456. 31295932 10.3390/ijerph16142456 PMC6678585

[7] World Health Organization. Malnutrition. World Health Organization. https://www.who.int/news -room/questions-and-answers/item/malnutrition. Accessed 5 Aug 2024.

[8] Psoter WJ, Spielman AL, Gebrian B, et al. Effect of childhood malnutrition on salivary flow and pH. Arch Oral Biol. 2008;53(3):231–7. 17983611 10.1016/j.archoralbio.2007.09.007 PMC2268214

[9] Lingstrom P, Moynihan P. Nutrition, saliva, and oral health. Nutrition. 2003;19(6):567–9. 12781864 10.1016/S0899-9007(03)00062-5

[10] American Academy of Pediatric Dentistry. Policy on early childhood caries (ECC): classifications, consequences, and preventive strategies. Pediatr Dent. 2014;36(6):50–2.

[11] Dimaisip-Nabuab J, Duijster D, Benzian H, et al. Nutritional status, dental caries and tooth eruption in children: a longitudinal study in Cambodia, Indonesia and Lao PDR. BMC Pediatr. 2018;18(1):300. 30217185 10.1186/s12887-018-1277-6 PMC6137874

[12] Kassebaum NJ, Bernabe E, Dahiya M, et al. Global burden of untreated caries: a systematic review and metaregression. J Dent Res. 2015;94(5):650–8. 25740856 10.1177/0022034515573272

[13] Sánchez-Pérez L, Sáenz-Martínez LP, Molina-Frechero N, et al. Body Mass Index and Dental Caries, a five-year Follow-Up study in Mexican Children. Int J Environ Res Public Health. 2021;18(14):7417. 34299868 10.3390/ijerph18147417 PMC8303166

[14] Olatosi OO, Alade AA, Naicker T, et al. Dental Caries Severity and Nutritional Status of Nigerian Preschool Children. JDR Clin Trans Res. 2022;7(2):154–62. 33764218 10.1177/23800844211002108 PMC8928415

[15] Li H, Li C, He X, et al. Dental caries status of preschool children in Chengdu and its correlation with growth and development and nutritional status. J Clin Stomatology. 2022;38(12):729–33.

[16] Manohar N, Hayen A, Fahey P, et al. Obesity and dental caries in early childhood: a systematic review and meta-analyses. Obes Rev. 2020;21(3):e12960. 31721413 10.1111/obr.12960

[17] Bafti LS, Hashemipour MA, Poureslami H, et al. Relationship between body Mass index and tooth decay in a Population of 3-6-year-old children in Iran. Int J Dent. 2015;2015:126530. 25788943 10.1155/2015/126530 PMC4348617

[18] Singh A, Purohit BM. Malnutrition and its Association with Dental Caries in the primary and permanent dentition: a systematic review and Meta-analysis. Pediatr Dent. 2020;42(6):418–26. 33369551

[19] Zuniga-Manriquez AG, Medina-Solis CE, Lara-Carrillo E et al. [Experience, prevalence and severity of dental caries and its association with nutritional status in Mexican infants 17–47 months. 23877810

[20] Nishimura M, Oda T, Kariya N, et al. Using a Caries Activity Test to Predict Caries Risk in Early Childhood. J Am Dent Association. 2008;139(1):63–71. 10.14219/jada.archive.2008.0022 18167387

[21] Yuan J, Xuan S, Wang J, et al. Study on caries prevalence status and caries activity detection in 3-year-old children in Haidian District, Beijing. Beijing Stomatology. 2017;25(03):166–8.

[22] Wang X. Study on the association between dietary quality and caries and caries activity in children aged 2–5 years old at a young age. Hebei Medical University; 2022.

[23] Fan Y, Yao Q, Liu Y, et al. Underlying causes and co-existence of Malnutrition and infections: an exceedingly common death risk in Cancer. Front Nutr. 2022;9:814095. 35284454 10.3389/fnut.2022.814095 PMC8906403

[24] Rakotonirainy NH, Razafindratovo V, Remonja CR, et al. Dietary diversity of 6- to 59-month-old children in rural areas of Moramanga and Morondava districts, Madagascar. PLoS ONE. 2018;13(7):e200235. 10.1371/journal.pone.0200235 PMC6044523 30005067

[25] Feng X. Oral health status of Chinese residents - A report of the fourth Chinese oral health epidemiological survey: 2018 18th Annual Conference on Preventive Dentistry of the Chinese Stomatological Association, Xi’an, Shaanxi, China, 2018.

[26] Petersen PE, Baez RJ, World HO. Oral health surveys: basic methods. Geneva: World Health Organization; 2013.

[27] Dikmen B. Icdas II criteria (international caries detection and assessment system). J Istanb Univ Fac Dent. 2015;49(3):63–72. 28955548 10.17096/jiufd.38691 PMC5573507

[28] Alkarimi HA, Watt RG, Pikhart H, et al. Dental caries and growth in school-age children. Pediatrics. 2014;133(3):e616–23. 24534405 10.1542/peds.2013-0846

[29] Anzar W, Qureshi A, Afaq A, et al. Association of Dental Caries and Anthropometric measures among Primary School Children. Children. 2021;8(3):223. 33805733 10.3390/children8030223 PMC8001750

[30] Institute of Nutrition and Food Safety. Chinese centre for Disease Control and Prevention, Hubei Provincial Centre for Disease Control and Prevention, Capital Institute of Paediatrics. et al. Anthropometric methods for population health monitoring. National Health and Family Planning Commission of the People’s Republic of China; 2013.

[31] National Health and Wellness Commission of the People’s Republic of China. Growth standards for children under 7 years of age (replacing WS/T 423–2013). National Health and Wellness Commission of the People’s Republic of China; 2023.

[32] McLean E, Cogswell M, Egli I, et al. Worldwide prevalence of anaemia, WHO Vitamin and Mineral Nutrition Information System, 1993–2005. Public Health Nutr. 2009;12(4):444–54. 18498676 10.1017/S1368980008002401

[33] Shen A, Bernabé E, Sabbah W. The bidirectional relationship between weight, height and dental caries among preschool children in China. PLoS ONE. 2019;14(4):e216227. 10.1371/journal.pone.0216227 PMC6490928 31039199

[34] Sheller B, Churchill SS, Williams BJ, et al. Body Mass Index of children with severe early childhood caries. Pediatr Dent. 2009;31(3):216–21. 19552226

[35] Renggli EP, Turton B, Sokal-Gutierrez K, et al. Stunting Malnutrition Associated with severe tooth decay in Cambodian toddlers. Nutrients. 2021;13(2):290. 33498508 10.3390/nu13020290 PMC7909538

[36] Mahboobi Z, Pakdaman A, Yazdani R, et al. Dietary free sugar and dental caries in children: a systematic review on longitudinal studies. Health Promot Perspect. 2021;11(3):271–80. 34660221 10.34172/hpp.2021.35 PMC8501477

[37] Ehizele AO, Ojehanon PI, Akhionbare O. Nutrition and oral health. Benin J Postgrad Med. 2009;11(1):885–96. 10.4314/bjpm.v11i1.48830

[38] Vieira KA, Rosa-Junior LS, Souza M, et al. Chronic malnutrition and oral health status in children aged 1 to 5 years: an observational study. Med (Baltim). 2020;99(18):e19595. 10.1097/MD.0000000000019595 PMC7440136 32358344

[39] So M, Ellenikiotis YA, Husby HM et al. Early Childhood Dental Caries, Mouth Pain, and Malnutrition in the Ecuadorian Amazon Region. Int J Environ Res Public Health, 2017,14(5). 10.3390/ijerph14050550 PMC5452000 28531148

[40] Penafiel D, Termote C, Lachat C, et al. Barriers to eating Traditional Foods Vary by Age Group in Ecuador with Biodiversity loss as a key issue. J Nutr Educ Behav. 2016;48(4):258–68. 26865357 10.1016/j.jneb.2015.12.003

[41] Hahn CL, Liewehr FR. Relationships between caries bacteria, host responses, and clinical signs and symptoms of pulpitis. J Endod. 2007;33(3):213–9. 17320699 10.1016/j.joen.2006.11.008

[42] Kumar S, Kroon J, Lalloo R, et al. Relationship between body mass index and dental caries in children, and the influence of socio-economic status. Int Dent J. 2017;67(2):91–7. 27747864 10.1111/idj.12259 PMC9376681

[43] Spreghini N, Cianfarani S, Spreghini MR, et al. Oral glucose effectiveness and metabolic risk in obese children and adolescents. Acta Diabetol. 2019;56(8):955–62. 30868315 10.1007/s00592-019-01303-y

[44] Tang R, Huang M, Huang S. Relationship between dental caries status and anemia in children with severe early childhood caries. Kaohsiung J Med Sci. 2013;29(6):330–6. 23684139 10.1016/j.kjms.2012.10.003 PMC11916650

[45] Paisi M, Kay E, Kaimi I, et al. Obesity and caries in four-to-six year old English children: a cross-sectional study. BMC Public Health. 2018;18(1):267. 29454320 10.1186/s12889-018-5156-8 PMC5816423

[46] Yao Y, Ren X, Song X, et al. The relationship between dental caries and obesity among primary school children aged 5 to 14 years. Nutr Hosp. 2014;30(1):60–5. 25137263 10.3305/nh.2014.30.1.7552

[47] Zhang M. A study on the correlation between caries, caries activity and growth and development of children aged 3–5 years old in Hohhot. Inner Mongolia Medical University; 2020.

[48] Zhang Q, Wang Y, Huang C. How do parental feeding knowledge and practices affect Chinese children’s Weight Status? Findings from multiple waves of CHNS. J Child Fam stud, 32(1), 123–34.

[49] World Health Organization. Micronutrients. https://www.who.int/health-topics/micronutrients. Accessed 5 Aug 2024.

[50] Hu K, Staiano AE. Trends in obesity prevalence among children and adolescents aged 2 to 19 years in the US from 2011 to 2020. JAMA Pediatr. 2022;176(10):1037–9. 35877133 10.1001/jamapediatrics.2022.2052 PMC9315946

[51] Krol DM, Whelan K. Maintaining and improving the oral health of Young Children. Pediatrics, 2023,151(1). 10.1542/peds.2022-060417 36530159

