# Supplementary material for: Study on the association between malnutrition, early childhood caries and caries activity among children aged 3–5 years
Source: BMC Oral Health. 2024 Sep 3;24:1035. doi: 10.1186/s12903-024-04802-9 (PMC11370079; doi:10.1186/s12903-024-04802-9)
Supplement: Supplementary file 1 — Supplementary Material 1: Growth Standards for Chinese Children Under 7 Years Old (Additional file 1). [file 12903_2024_4802_MOESM1_ESM.docx]

| **Growth standards for children under 7 years of age**  **Standard deviation assessment method for child nutritional status** | | | | |
| --- | --- | --- | --- | --- |
| standard deviation method | Evaluation indicators | | | |
|  | WAZ(Weight-for-age Z-score) | HAZ(Height-for-age Z-score) | HWZ(height-for- weight Z-score | BAZ(BMI-for-age Z-score) |
| ≥+3 SD |  |  | Severe obese | Severe obese |
| +2 SD≤-<+3 SD |  |  | obese | obese |
| +1 SD≤-<+2 SD | - |  | overweight | overweight |
| -1 SD≤-<+1 SD | - |  |  |  |
| -2 SD≤-<-1 SD |  |  |  |  |
| 3 SD≤-<-2 SD | low weight | stunting | wasting | wasting |
| <-3 SD | Severe low weight | Severe stunting | severe wasting | severe wasting |
