# Supplementary material for: Study on the association between malnutrition, early childhood caries and caries activity among children aged 3–5 years
Source: BMC Oral Health. 2024 Sep 3;24:1035. doi: 10.1186/s12903-024-04802-9 (PMC11370079; doi:10.1186/s12903-024-04802-9)
Supplement: Supplementary file 2 — Supplementary Material 2: Evaluation table for standard deviation (Additional file 2). [file 12903_2024_4802_MOESM2_ESM.pdf]

Table B.1 Standard deviation values of Weight-for-age for boys under 7 years of age

Unit in kilograms

age	-3 *SD*	-2 *SD*	-1 *SD*	median	+1 *SD*	+2 *SD*	+3 *SD*
0 month	2.4	2.7	3.1	3.5	3.9	4.3	4.7
1 month	3.2	3.6	4.1	4.6	5.1	5.6	6.2
2 months	4.1	4.6	5.2	5.8	6.5	7.2	8.0
3 months	4.9	5.5	6.1	6.8	7.6	8.4	9.3
4 months	5.4	6.0	6.7	7.5	8.3	9.3	10.3
5 months	5.8	6.5	7.2	8.0	8.9	9.9	11.1
6 months	6.1	6.8	7.6	8.4	9.4	10.5	11.7
7 months	6.4	7.1	7.9	8.8	9.8	10.9	12.1
8 months	6.7	7.4	8.2	9.1	10.1	11.3	12.6
9 months	6.9	7.6	8.4	9.4	10.4	11.6	12.9
10 months	7.1	7.8	8.7	9.6	10.7	11.9	13.3
11 months	7.2	8.0	8.9	9.8	10.9	12.2	13.6
1 year	7.4	8.2	9.1	10.1	11.2	12.4	13.9
1year and 1month	7.5	8.3	9.2	10.3	11.4	12.7	14.1
1year and 2months	7.7	8.5	9.4	10.5	11.6	12.9	14.4
1year and 3months	7.8	8.7	9.6	10.7	11.8	13.2	14.7
1year and 4months	8.0	8.8	9.8	10.9	12.1	13.4	15.0
1year and 5months	8.2	9.0	10.0	11.1	12.3	13.7	15.3
1year and 6months	8.3	9.2	10.2	11.3	12.5	14.0	15.6
1year and 7months	8.5	9.4	10.4	11.5	12.8	14.2	15.9
1 year and 8months	8.6	9.5	10.6	11.7	13.0	14.5	16.2
1year and 9months	8.8	9.7	10.8	11.9	13.3	14.8	16.5
1year and 10months	9.0	9.9	11.0	12.2	13.5	15.0	16.8
1year and 11months	9.1	10.1	11.1	12.4	13.7	15.3	17.1
2years	9.3	10.2	11.3	12.6	14.0	15.6	17.4
2years and 3months	9.7	10.7	11.8	13.1	14.6	16.3	18.2
2years and 6months	10.1	11.1	12.3	13.7	15.2	17.0	19.0
2years and 9months	10.4	11.5	12.7	14.2	15.8	17.6	19.8
3years	10.8	11.9	13.2	14.6	16.3	18.3	20.5
3years and 3months	11.1	12.3	13.6	15.2	16.9	19.0	21.3
3years and 6months	11.5	12.7	14.1	15.7	17.5	19.7	22.2
3years and 9months	11.8	13.1	14.5	16.2	18.1	20.4	23.0
4years	12.2	13.5	15.0	16.7	18.8	21.1	23.9
4years and 3months	12.5	13.9	15.5	17.3	19.4	21.9	24.9
4years and 6months	12.8	14.3	15.9	17.9	20.1	22.7	25.9
4years and 9months	13.2	14.7	16.4	18.4	20.8	23.6	27.0
5 years	13.6	15.1	16.9	19.1	21.6	24.5	28.1
5years and 3months	13.9	15.6	17.5	19.7	22.3	25.5	29.3

Table B.1 continued

Unit in kilograms

age	-3 *SD*	-2 *SD*	-1 *SD*	median	+1 *SD*	+2 *SD*	+3 *SD*
5years and 6months	14.3	16.0	18.0	20.3	23.1	26.4	30.5
5years and 9months	14.6	16.4	18.5	21.0	23.9	27.4	31.7
6 years	14.9	16.8	19.0	21.6	24.7	28.4	32.9
6years and 3months	15.3	17.2	19.5	22.2	25.5	29.4	34.1
6years and 6months	15.5	17.6	20.0	22.8	26.2	30.3	35.3
6years and 9months	15.8	17.9	20.4	23.4	26.9	31.2	36.4
Note: Age is in whole months or years.							

Table B.2 Standard deviation values of Weight-for-age for girls under 7 years of age

Unit in kilograms

age	-3 *SD*	-2 *SD*	-1 *SD*	median	+1 *SD*	+2 *SD*	+3 *SD*
0 month	2.3	2.6	3.0	3.3	3.7	4.1	4.6
1 month	3.0	3.4	3.8	4.3	4.8	5.3	5.9
2 months	3.8	4.3	4.8	5.4	6.0	6.7	7.4
3 months	4.5	5.0	5.6	6.2	6.9	7.7	8.6
4 months	5.0	5.5	6.2	6.9	7.7	8.6	9.6
5 months	5.4	6.0	6.6	7.4	8.2	9.2	10.3
6 months	5.7	6.3	7.0	7.8	8.7	9.7	10.9
7 months	6.0	6.6	7.3	8.1	9.1	10.2	11.5
8 months	6.2	6.9	7.6	8.4	9.4	10.6	11.9
9 months	6.4	7.1	7.8	8.7	9.7	10.9	12.3
10 months	6.6	7.3	8.1	9.0	10.0	11.2	12.7
11 months	6.8	7.5	8.3	9.2	10.3	11.5	13.0
1year	6.9	7.7	8.5	9.4	10.5	11.8	13.3
1year and 1month	7.1	7.8	8.7	9.6	10.7	12.1	13.6
1year and 2months	7.3	8.0	8.8	9.8	11.0	12.3	13.9
1year and 3months	7.4	8.2	9.0	10.0	11.2	12.6	14.2
1year and 4months	7.6	8.3	9.2	10.3	11.5	12.9	14.5
1year and 5months	7.7	8.5	9.4	10.5	11.7	13.1	14.9
1year and 6months	7.9	8.7	9.6	10.7	11.9	13.4	15.2
1year and 7months	8.1	8.9	9.8	10.9	12.2	13.7	15.5
1year and 8months	8.2	9.0	10.0	11.1	12.4	13.9	15.8
1year and 9months	8.4	9.2	10.2	11.3	12.6	14.2	16.1
1year and 10months	8.5	9.4	10.4	11.5	12.9	14.5	16.4
1year and 11months	8.7	9.5	10.6	11.7	13.1	14.8	16.7
2years	8.8	9.7	10.7	11.9	13.3	15.0	17.0
2years and 3months	9.2	10.1	11.2	12.5	14.0	15.8	17.9
2years and 6months	9.6	10.6	11.7	13.0	14.6	16.5	18.7

Table B.2 continued

Unit in kilograms

age	-3 *SD*	-2 *SD*	-1 *SD*	median	+1 *SD*	+2 *SD*	+3 *SD*
2years and 9months	10.0	11.0	12.2	13.6	15.2	17.2	19.6
3years	10.3	11.4	12.6	14.1	15.9	17.9	20.5
3years and 3months	10.7	11.8	13.1	14.7	16.5	18.7	21.3
3years and 6months	11.1	12.2	13.6	15.2	17.1	19.4	22.2
3years and 9months	11.4	12.6	14.0	15.7	17.7	20.1	23.0
4years	11.7	13.0	14.5	16.2	18.3	20.8	23.8
4years and 3months	12.0	13.3	14.9	16.7	18.9	21.5	24.6
4years and 6months	12.3	13.7	15.3	17.2	19.5	22.2	25.5
4years and 9months	12.7	14.1	15.8	17.8	20.2	23.0	26.4
5years	13.0	14.5	16.3	18.4	20.9	23.8	27.4
5years and 3months	13.3	14.9	16.8	19.0	21.6	24.7	28.4
5years and 6months	13.7	15.3	17.3	19.6	22.3	25.5	29.5
5years and 9months	14.0	15.7	17.8	20.2	23.0	26.4	30.5
6years	14.3	16.1	18.2	20.7	23.7	27.3	31.5
6years and 3months	14.5	16.4	18.7	21.3	24.4	28.1	32.6
6years and 6months	14.8	16.8	19.1	21.8	25.1	28.9	33.6
6years and 9months	15.0	17.1	19.5	22.4	25.8	29.8	34.6
Note: Age is in whole months or years.							

Table B.3 Standard deviation values of Height-for-age for boys under 7 years of age

Unit in centimetres

age	-3 *SD*	-2 *SD*	-1 *SD*	median	+1 *SD*	+2 *SD*	+3 *SD*
0 month	45.4	47.3	49.2	51.2	53.1	55.0	56.9
1 month	49.1	51.1	53.1	55.1	57.2	59.2	61.2
2 months	52.6	54.7	56.8	59.0	61.1	63.2	65.4
3 months	55.5	57.8	60.0	62.2	64.4	66.6	68.9
4 months	58.0	60.3	62.5	64.8	67.1	69.4	71.7
5 months	59.9	62.3	64.6	66.9	69.3	71.6	74.0
6 months	61.6	64.0	66.3	68.7	71.1	73.5	75.9
7 months	63.0	65.4	67.9	70.3	72.7	75.1	77.6
8 months	64.3	66.8	69.3	71.7	74.2	76.7	79.1
9 months	65.5	68.0	70.5	73.1	75.6	78.1	80.6
10 months	66.7	69.2	71.8	74.3	76.9	79.4	82.0
11 months	67.8	70.3	72.9	75.5	78.1	80.7	83.3
1year	68.8	71.4	74.1	76.7	79.3	81.9	84.6
1year and 1month	69.8	72.5	75.1	77.8	80.5	83.1	85.8
1year and 2months	70.8	73.5	76.2	78.9	81.6	84.3	87.0
1year and 3months	71.7	74.5	77.2	80.0	82.7	85.5	88.2

Table B.3 continued

Unit in centimetres

age	-3 *SD*	-2 *SD*	-1 *SD*	median	+1 *SD*	+2 *SD*	+3 *SD*
1year and 4months	72.7	75.5	78.2	81.0	83.8	86.6	89.4
1year and 5months	73.6	76.4	79.2	82.1	84.9	87.7	90.5
1year and 6months	74.5	77.4	80.2	83.1	86.0	88.8	91.7
1year and 7months	75.4	78.3	81.2	84.1	87.0	89.9	92.8
1year and 8months	76.3	79.2	82.2	85.1	88.0	91.0	93.9
1year and 9months	77.1	80.1	83.1	86.1	89.1	92.0	95.0
1year and 10months	78.0	81.0	84.0	87.0	90.1	93.1	96.1
1year and 11months	78.8	81.9	84.9	88.0	91.0	94.1	97.2
2years	78.9	82.0	85.1	88.2	91.3	94.4	97.5
2years and 3months	81.2	84.4	87.6	90.8	94.0	97.2	100.4
2years and 6months	83.3	86.6	89.9	93.2	96.5	99.8	103.1
2years and 9months	85.2	88.6	92.0	95.4	98.8	102.2	105.6
3years	87.0	90.5	94.0	97.5	101.0	104.5	108.0
3years and 3months	88.6	92.2	95.9	99.5	103.1	106.7	110.3
3years and 6months	90.3	93.9	97.6	101.3	105.0	108.7	112.4
3years and 9months	91.8	95.6	99.4	103.1	106.9	110.7	114.5
4years	93.3	97.2	101.0	104.9	108.8	112.6	116.5
4years and 3months	94.8	98.8	102.7	106.6	110.6	114.5	118.5
4years and 6months	96.3	100.3	104.4	108.4	112.4	116.5	120.5
4years and 9months	97.8	102.0	106.1	110.2	114.3	118.4	122.5
5years	99.4	103.6	107.8	112.0	116.2	120.4	124.6
5years and 3months	100.9	105.2	109.5	113.7	118.0	122.3	126.6
5years and 6months	102.3	106.7	111.1	115.5	119.8	124.2	128.6
5years and 9months	103.8	108.2	112.7	117.1	121.6	126.1	130.5
6years	105.2	109.7	114.3	118.8	123.3	127.9	132.4
6years and 3months	106.5	111.2	115.8	120.4	125.0	129.7	134.3
6years and 6months	107.9	112.6	117.3	122.0	126.7	131.4	136.1
6years and 9months	109.2	113.9	118.7	123.5	128.3	133.1	137.9

Table B.4 Standard deviation values of Height-for-age for girls under 7 years of age

Unit in kilograms

age	-3 *SD*	-2 *SD*	-1 *SD*	median	+1 *SD*	+2 *SD*	+3 *SD*
0month	44.7	46.6	48.4	50.3	52.2	54.1	55.9
1month	48.2	50.1	52.1	54.1	56.1	58.1	60.0
2months	51.5	53.5	55.6	57.7	59.8	61.9	63.9
3months	54.3	56.4	58.6	60.8	62.9	65.1	67.2
4months	56.6	58.8	61.0	63.3	65.5	67.7	69.9

Table B.4 continued

Unit in centimetres

age	-3 *SD*	-2 *SD*	-1 *SD*	median	+1 *SD*	+2 *SD*	+3 *SD*
5months	58.5	60.7	63.0	65.3	67.6	69.9	72.2
6months	60.1	62.4	64.7	67.1	69.4	71.7	74.1
7months	61.5	63.9	66.3	68.7	71.0	73.4	75.8
8months	62.8	65.3	67.7	70.1	72.5	75.0	77.4
9months	64.1	66.5	69.0	71.5	73.9	76.4	78.9
10months	65.3	67.8	70.3	72.8	75.3	77.8	80.3
11months	66.4	68.9	71.5	74.0	76.6	79.1	81.7
1year	67.5	70.1	72.6	75.2	77.8	80.4	83.0
1year and 1month	68.5	71.1	73.8	76.4	79.0	81.7	84.3
1year and 2months	69.5	72.2	74.9	77.5	80.2	82.9	85.6
1year and 3months	70.5	73.2	75.9	78.6	81.4	84.1	86.8
1year and 4months	71.5	74.2	77.0	79.7	82.5	85.2	88.0
1year and 5months	72.4	75.2	78.0	80.8	83.6	86.4	89.2
1year and 6months	73.3	76.2	79.0	81.9	84.7	87.5	90.4
1year and 7months	74.3	77.1	80.0	82.9	85.8	88.6	91.5
1year and 8months	75.1	78.1	81.0	83.9	86.8	89.7	92.6
1year and 9months	76.0	79.0	81.9	84.9	87.8	90.8	93.7
1year and 10months	76.9	79.9	82.8	85.8	88.8	91.8	94.8
1year and 11months	77.7	80.7	83.7	86.8	89.8	92.8	95.9
2years	77.8	80.8	83.9	87.0	90.1	93.1	96.2
2years and 3months	80.0	83.2	86.4	89.5	92.7	95.9	99.1
2years and 6months	82.1	85.3	88.6	91.9	95.2	98.5	101.7
2years and 9months	84.0	87.3	90.7	94.1	97.5	100.9	104.2
3years	85.8	89.3	92.7	96.2	99.7	103.2	106.6
3year and 3months	87.5	91.1	94.6	98.2	101.8	105.3	108.9
3year and 6months	89.1	92.8	96.4	100.1	103.7	107.4	111.0
3year and 9months	90.7	94.4	98.2	101.9	105.6	109.4	113.1
4years	92.2	96.0	99.8	103.7	107.5	111.3	115.1
4years and 3months	93.7	97.6	101.5	105.4	109.3	113.2	117.2
4years and 6months	95.2	99.2	103.2	107.2	111.2	115.2	119.2
4years and 9months	96.8	100.8	104.9	109.0	113.1	117.2	121.2
5years	98.3	102.5	106.6	110.8	115.0	119.1	123.3
5years and 3months	99.8	104.1	108.3	112.6	116.8	121.1	125.3
5years and 6months	101.2	105.6	109.9	114.3	118.6	123.0	127.3
5years and 9months	102.6	107.1	111.5	115.9	120.4	124.8	129.2
6years	104.0	108.5	113.0	117.5	122.0	126.5	131.0
6years and 3months	105.3	109.9	114.5	119.1	123.7	128.2	132.8
6years and6months	106.6	111.3	115.9	120.6	125.3	129.9	134.6

T a b l e B. 4 continued

Unit in centimetres

age	-3 *SD*	-2 *SD*	-1 *SD*	median	+1 *SD*	+2 *SD*	+3 *SD*
6years and 9months	107.9	112.6	117.3	122.1	126.8	131.6	136.3

T a b l e B. 5 Standard deviation values of length-for-weight for boys aged 0–2 years

Unit in kilograms

length of body (centimetres)	-3 *SD*	-2 *SD*	-1 *SD*	median	+1 *SD*	+2 *SD*	+3 *SD*
45	1.8	2.0	2.1	2.3	2.5	2.8	3.0
46	1.9	2.1	2.3	2.5	2.7	3.0	3.3
47	2.1	2.3	2.5	2.7	2.9	3.2	3.5
48	2.2	2.4	2.6	2.9	3.2	3.5	3.8
49	2.4	2.6	2.8	3.1	3.4	3.7	4.0
50	2.5	2.8	3.0	3.3	3.6	3.9	4.3
51	2.7	3.0	3.2	3.5	3.8	4.2	4.6
52	2.9	3.2	3.4	3.8	4.1	4.5	4.9
53	3.1	3.4	3.7	4.0	4.4	4.8	5.3
54	3.3	3.6	3.9	4.3	4.7	5.2	5.7
55	3.6	3.9	4.2	4.6	5.0	5.5	6.1
56	3.8	4.1	4.5	4.9	5.3	5.9	6.5
57	4.0	4.4	4.8	5.2	5.7	6.2	6.9
58	4.3	4.6	5.0	5.5	6.0	6.6	7.3
59	4.5	4.9	5.3	5.8	6.3	7.0	7.7
60	4.7	5.1	5.6	6.1	6.7	7.3	8.1
61	5.0	5.4	5.9	6.4	7.0	7.7	8.5
62	5.2	5.6	6.1	6.7	7.3	8.0	8.8
63	5.4	5.9	6.4	6.9	7.6	8.3	9.2
64	5.7	6.1	6.6	7.2	7.9	8.7	9.6
65	5.9	6.3	6.9	7.5	8.2	9.0	9.9
66	6.1	6.6	7.1	7.7	8.5	9.3	10.3
67	6.3	6.8	7.3	8.0	8.7	9.6	10.6
68	6.5	7.0	7.6	8.2	9.0	9.9	10.9
69	6.7	7.2	7.8	8.5	9.2	10.1	11.2
70	6.9	7.4	8.0	8.7	9.5	10.4	11.5
71	7.1	7.6	8.2	8.9	9.7	10.7	11.8
72	7.3	7.8	8.4	9.1	10.0	10.9	12.1
73	7.4	8.0	8.6	9.3	10.2	11.2	12.3
74	7.6	8.2	8.8	9.5	10.4	11.4	12.6
75	7.8	8.3	9.0	9.7	10.6	11.6	12.9

Table B.5 continued

Unit in kilograms

length of body (centimetres)	-3 *SD*	-2 *SD*	-1 *SD*	median	+1 *SD*	+2 *SD*	+3 *SD*
76	7.9	8.5	9.2	9.9	10.8	11.9	13.1
77	8.1	8.7	9.4	10.1	11.0	12.1	13.3
78	8.3	8.9	9.5	10.3	11.2	12.3	13.6
79	8.4	9.0	9.7	10.5	11.4	12.5	13.8
80	8.6	9.2	9.9	10.7	11.6	12.7	14.1
81	8.8	9.4	10.1	10.9	11.8	13.0	14.3
82	8.9	9.6	10.3	11.1	12.0	13.2	14.5
83	9.1	9.7	10.5	11.3	12.3	13.4	14.8
84	9.3	9.9	10.6	11.5	12.5	13.6	15.0
85	9.5	10.1	10.8	11.7	12.7	13.9	15.3
86	9.6	10.3	11.0	11.9	12.9	14.1	15.5
87	9.8	10.5	11.2	12.1	13.1	14.4	15.8
88	10.0	10.7	11.4	12.3	13.4	14.6	16.1
89	10.2	10.9	11.7	12.6	13.6	14.9	16.4
90	10.4	11.1	11.9	12.8	13.8	15.1	16.6
91	10.6	11.3	12.1	13.0	14.1	15.4	16.9
92	10.8	11.5	12.3	13.2	14.3	15.6	17.2
93	11.0	11.7	12.5	13.4	14.6	15.9	17.5
94	11.2	11.9	12.7	13.7	14.8	16.1	17.8
95	11.4	12.1	12.9	13.9	15.1	16.4	18.1
96	11.6	12.3	13.2	14.2	15.3	16.7	18.4
97	11.8	12.5	13.4	14.4	15.6	17.0	18.7
98	12.0	12.8	13.6	14.7	15.9	17.3	19.0
99	12.2	13.0	13.9	14.9	16.1	17.6	19.3
100	12.5	13.2	14.1	15.2	16.4	17.9	19.7

Table B.6 Standard deviation values of length-for-weight for girls aged 0-2 years

Unit in kilograms

length of body (centimetres)	-3 *SD*	-2 *SD*	-1 *SD*	median	+1 *SD*	+2 *SD*	+3 *SD*
45	1.8	2.0	2.2	2.3	2.6	2.8	3.1
46	2.0	2.1	2.3	2.5	2.8	3.0	3.3
47	2.1	2.3	2.5	2.7	3.0	3.2	3.5
48	2.2	2.4	2.7	2.9	3.2	3.5	3.8
49	2.4	2.6	2.8	3.1	3.4	3.7	4.0
50	2.6	2.8	3.0	3.3	3.6	3.9	4.3

Table B.6 continued

Unit in kilograms

length of body (centimetres)	-3 *SD*	-2 *SD*	-1 *SD*	median	+1 *SD*	+2 *SD*	+3 *SD*
51	2.7	3.0	3.2	3.5	3.8	4.2	4.6
52	2.9	3.2	3.5	3.8	4.1	4.5	5.0
53	3.2	3.4	3.7	4.0	4.4	4.8	5.3
54	3.4	3.6	4.0	4.3	4.7	5.2	5.7
55	3.6	3.9	4.2	4.6	5.0	5.5	6.1
56	3.8	4.1	4.5	4.9	5.3	5.8	6.4
57	4.0	4.4	4.7	5.1	5.6	6.2	6.8
58	4.3	4.6	5.0	5.4	5.9	6.5	7.2
59	4.5	4.8	5.2	5.7	6.2	6.8	7.6
60	4.7	5.1	5.5	6.0	6.5	7.2	7.9
61	4.9	5.3	5.7	6.2	6.8	7.5	8.3
62	5.1	5.5	6.0	6.5	7.1	7.8	8.6
63	5.3	5.7	6.2	6.8	7.4	8.1	9.0
64	5.6	6.0	6.5	7.0	7.7	8.4	9.3
65	5.8	6.2	6.7	7.3	7.9	8.7	9.7
66	6.0	6.4	6.9	7.5	8.2	9.0	10.0
67	6.1	6.6	7.1	7.7	8.4	9.3	10.3
68	6.3	6.8	7.3	8.0	8.7	9.5	10.6
69	6.5	7.0	7.5	8.2	8.9	9.8	10.9
70	6.7	7.2	7.7	8.4	9.1	10.1	11.1
71	6.9	7.4	7.9	8.6	9.4	10.3	11.4
72	7.0	7.5	8.1	8.8	9.6	10.5	11.7
73	7.2	7.7	8.3	9.0	9.8	10.8	11.9
74	7.4	7.9	8.5	9.2	10.0	11.0	12.2
75	7.5	8.1	8.7	9.4	10.2	11.2	12.4
76	7.7	8.2	8.9	9.6	10.4	11.4	12.7
77	7.9	8.4	9.0	9.8	10.6	11.6	12.9
78	8.0	8.6	9.2	9.9	10.8	11.9	13.1
79	8.2	8.7	9.4	10.1	11.0	12.1	13.4
80	8.3	8.9	9.6	10.3	11.2	12.3	13.6
81	8.5	9.1	9.7	10.5	11.4	12.5	13.9
82	8.7	9.3	9.9	10.7	11.6	12.8	14.1
83	8.9	9.4	10.1	10.9	11.9	13.0	14.4
84	9.0	9.6	10.3	11.1	12.1	13.2	14.6
85	9.2	9.8	10.5	11.3	12.3	13.5	14.9
86	9.4	10.0	10.7	11.6	12.5	13.7	15.2
87	9.6	10.2	10.9	11.8	12.8	14.0	15.4

Table B.6 continued

Unit in kilograms

length of body (centimetres)	-3 *SD*	-2 *SD*	-1 *SD*	median	+1 *SD*	+2 *SD*	+3 *SD*
88	9.8	10.4	11.1	12.0	13.0	14.2	15.7
89	9.9	10.6	11.3	12.2	13.2	14.5	16.0
90	10.1	10.8	11.5	12.4	13.5	14.7	16.3
91	10.3	11.0	11.8	12.7	13.7	15.0	16.6
92	10.5	11.2	12.0	12.9	14.0	15.3	16.9
93	10.7	11.4	12.2	13.1	14.2	15.6	17.2
94	10.9	11.6	12.4	13.4	14.5	15.8	17.5
95	11.1	11.8	12.7	13.6	14.8	16.1	17.8
96	11.3	12.0	12.9	13.9	15.0	16.4	18.2
97	11.5	12.3	13.1	14.1	15.3	16.8	18.5
98	11.7	12.5	13.4	14.4	15.6	17.1	18.9
99	12.0	12.7	13.6	14.7	15.9	17.4	19.2
100	12.2	13.0	13.9	14.9	16.2	17.7	19.6

Table B.7 Standard deviation values of length-for-weight for boys aged 2–7 years

Unit in kilograms

length of body (centimetres)	-3 *SD*	-2 *SD*	-1 *SD*	median	+1 *SD*	+2 *SD*	+3 *SD*
75	7.9	8.5	9.1	9.9	10.8	11.8	13.0
76	8.1	8.6	9.3	10.1	11.0	12.0	13.3
77	8.2	8.8	9.5	10.3	11.2	12.2	13.5
78	8.4	9.0	9.7	10.5	11.4	12.5	13.8
79	8.5	9.1	9.8	10.6	11.6	12.7	14.0
80	8.7	9.3	10.0	10.8	11.8	12.9	14.2
81	8.9	9.5	10.2	11.0	12.0	13.1	14.5
82	9.0	9.7	10.4	11.2	12.2	13.3	14.7
83	9.2	9.9	10.6	11.4	12.4	13.6	15.0
84	9.4	10.0	10.8	11.6	12.6	13.8	15.2
85	9.6	10.2	11.0	11.8	12.8	14.0	15.5
86	9.8	10.4	11.2	12.1	13.1	14.3	15.7
87	10.0	10.6	11.4	12.3	13.3	14.5	16.0
88	10.1	10.8	11.6	12.5	13.5	14.8	16.3
89	10.3	11.0	11.8	12.7	13.8	15.0	16.6
90	10.5	11.2	12.0	12.9	14.0	15.3	16.8
91	10.7	11.4	12.2	13.2	14.2	15.5	17.1
92	10.9	11.6	12.4	13.4	14.5	15.8	17.4

Table B.7 continued

Unit in kilograms

length of body (centimetres)	-3 *SD*	-2 *SD*	-1 *SD*	median	+1 *SD*	+2 *SD*	+3 *SD*
93	11.1	11.8	12.6	13.6	14.7	16.1	17.7
94	11.3	12.0	12.9	13.8	15.0	16.3	18.0
95	11.5	12.2	13.1	14.1	15.2	16.6	18.3
96	11.7	12.5	13.3	14.3	15.5	16.9	18.6
97	11.9	12.7	13.6	14.6	15.8	17.2	18.9
98	12.2	12.9	13.8	14.8	16.1	17.5	19.2
99	12.4	13.2	14.1	15.1	16.3	17.8	19.6
100	12.6	13.4	14.3	15.4	16.6	18.1	19.9
101	12.9	13.7	14.6	15.7	16.9	18.5	20.3
102	13.1	13.9	14.9	16.0	17.3	18.8	20.7
103	13.3	14.2	15.1	16.3	17.6	19.2	21.1
104	13.6	14.4	15.4	16.6	17.9	19.5	21.5
105	13.8	14.7	15.7	16.8	18.2	19.9	21.9
106	14.0	14.9	16.0	17.1	18.6	20.2	22.3
107	14.3	15.2	16.2	17.4	18.9	20.6	22.7
108	14.5	15.4	16.5	17.8	19.2	21.0	23.2
109	14.7	15.7	16.8	18.1	19.6	21.4	23.7
110	15.0	15.9	17.1	18.4	20.0	21.8	24.2
111	15.2	16.2	17.4	18.7	20.3	22.3	24.7
112	15.4	16.5	17.7	19.1	20.7	22.8	25.2
113	15.7	16.7	18.0	19.4	21.2	23.3	25.8
114	15.9	17.0	18.3	19.8	21.6	23.8	26.5
115	16.2	17.3	18.6	20.2	22.1	24.3	27.2
116	16.4	17.6	19.0	20.6	22.6	24.9	27.9
117	16.7	17.9	19.3	21.0	23.1	25.6	28.7
118	16.9	18.2	19.7	21.5	23.6	26.2	29.5
119	17.2	18.5	20.1	21.9	24.2	26.9	30.4
120	17.5	18.9	20.5	22.4	24.8	27.6	31.3
121	17.8	19.2	20.9	22.9	25.4	28.4	32.2
122	18.0	19.5	21.3	23.4	26.0	29.2	33.2
123	18.3	19.9	21.7	23.9	26.6	30.0	34.3
124	18.6	20.2	22.1	24.4	27.3	30.8	35.3
125	18.9	20.6	22.6	25.0	27.9	31.6	36.4
126	19.2	20.9	23.0	25.5	28.6	32.5	37.5
127	19.4	21.3	23.4	26.0	29.3	33.4	38.6
128	19.7	21.6	23.9	26.6	30.0	34.2	39.8
129	20.0	21.9	24.3	27.1	30.7	35.1	40.9

Table B.7 continued

Unit in kilograms

length of body (centimetres)	-3 *SD*	-2 *SD*	-1 *SD*	median	+1 *SD*	+2 *SD*	+3 *SD*
130	20.2	22.3	24.7	27.7	31.3	36.0	42.1

Table B.8 Standard deviation values of length-for-weight for girls aged 2-7 years

Unit in kilograms

length of body (centimetres)	-3 *SD*	-2 *SD*	-1 *SD*	median	+1 *SD*	+2 *SD*	+3 *SD*
75	7.7	8.2	8.8	9.5	10.4	11.4	12.6
76	7.8	8.4	9.0	9.7	10.6	11.6	12.8
77	8.0	8.5	9.2	9.9	10.8	11.8	13.1
78	8.1	8.7	9.3	10.1	11.0	12.0	13.3
79	8.3	8.9	9.5	10.3	11.2	12.2	13.5
80	8.5	9.0	9.7	10.5	11.4	12.5	13.8
81	8.6	9.2	9.9	10.7	11.6	12.7	14.0
82	8.8	9.4	10.1	10.9	11.8	12.9	14.3
83	9.0	9.6	10.3	11.1	12.0	13.2	14.5
84	9.2	9.8	10.5	11.3	12.2	13.4	14.8
85	9.3	10.0	10.7	11.5	12.5	13.6	15.1
86	9.5	10.1	10.9	11.7	12.7	13.9	15.4
87	9.7	10.3	11.1	11.9	12.9	14.1	15.6
88	9.9	10.5	11.3	12.1	13.2	14.4	15.9
89	10.1	10.7	11.5	12.4	13.4	14.7	16.2
90	10.3	10.9	11.7	12.6	13.6	14.9	16.5
91	10.4	11.1	11.9	12.8	13.9	15.2	16.8
92	10.6	11.3	12.1	13.1	14.2	15.5	17.1
93	10.8	11.5	12.4	13.3	14.4	15.8	17.4
94	11.0	11.8	12.6	13.5	14.7	16.0	17.7
95	11.2	12.0	12.8	13.8	15.0	16.4	18.1
96	11.5	12.2	13.1	14.1	15.2	16.7	18.4
97	11.7	12.4	13.3	14.3	15.5	17.0	18.8
98	11.9	12.7	13.6	14.6	15.8	17.3	19.1
99	12.1	12.9	13.8	14.9	16.1	17.6	19.5
100	12.3	13.1	14.1	15.1	16.4	18.0	19.9
101	12.5	13.4	14.3	15.4	16.7	18.3	20.2
102	12.8	13.6	14.6	15.7	17.0	18.6	20.6
103	13.0	13.8	14.8	16.0	17.3	19.0	21.0
104	13.2	14.1	15.1	16.3	17.7	19.3	21.4

Table B.8 continued

Unit in kilograms

length of body (centimetres)	-3 *SD*	-2 *SD*	-1 *SD*	median	+1 *SD*	+2 *SD*	+3 *SD*
105	13.4	14.3	15.3	16.5	18.0	19.7	21.8
106	13.6	14.5	15.6	16.8	18.3	20.1	22.2
107	13.8	14.8	15.9	17.1	18.6	20.4	22.6
108	14.1	15.0	16.1	17.4	19.0	20.8	23.1
109	14.3	15.3	16.4	17.8	19.3	21.2	23.6
110	14.5	15.5	16.7	18.1	19.7	21.7	24.1
111	14.7	15.8	17.0	18.4	20.1	22.1	24.6
112	15.0	16.1	17.3	18.8	20.5	22.6	25.1
113	15.2	16.3	17.6	19.1	20.9	23.1	25.7
114	15.4	16.6	17.9	19.5	21.3	23.6	26.3
115	15.7	16.9	18.3	19.9	21.8	24.1	27.0
116	15.9	17.2	18.6	20.3	22.3	24.7	27.6
117	16.2	17.5	18.9	20.7	22.7	25.3	28.4
118	16.4	17.7	19.3	21.1	23.2	25.9	29.1
119	16.7	18.1	19.6	21.5	23.8	26.5	29.9
120	16.9	18.4	20.0	22.0	24.3	27.1	30.7
121	17.2	18.7	20.4	22.4	24.9	27.8	31.5
122	17.5	19.0	20.8	22.9	25.4	28.5	32.4
123	17.7	19.3	21.2	23.4	26.0	29.2	33.2
124	18.0	19.6	21.6	23.8	26.6	29.9	34.1
125	18.3	20.0	22.0	24.3	27.2	30.7	35.0
126	18.5	20.3	22.4	24.8	27.8	31.4	36.0
127	18.8	20.6	22.8	25.3	28.4	32.2	36.9
128	19.1	20.9	23.2	25.8	29.0	32.9	37.8
129	19.3	21.3	23.5	26.3	29.6	33.7	38.8
130	19.6	21.6	23.9	26.8	30.2	34.4	39.7

Table B.9 Standard deviation values of BMI-for-age for boys under 7 years of age

In kilograms per square metre

age	-3 *SD*	-2 *SD*	-1 *SD*	median	+1 *SD*	+2 *SD*	+3 *SD*
0month	10.2	11.1	12.1	13.2	14.4	15.7	17.1
1months	11.8	12.9	14.0	15.1	16.4	17.7	19.2
2months	13.1	14.2	15.4	16.7	18.1	19.7	21.4
3months	13.6	14.8	16.0	17.4	19.0	20.8	22.7
4months	13.9	15.0	16.3	17.8	19.4	21.2	23.3
5months	14.0	15.1	16.4	17.9	19.5	21.4	23.5

Table B.9 continued

In kilograms per square metre

age	-3 *SD*	-2 *SD*	-1 *SD*	median	+1 *SD*	+2 *SD*	+3 *SD*
6months	14.1	15.2	16.4	17.9	19.5	21.4	23.5
7months	14.1	15.2	16.4	17.8	19.4	21.3	23.4
8months	14.1	15.1	16.3	17.7	19.3	21.1	23.2
9months	14.0	15.1	16.2	17.6	19.1	20.9	23.0
10months	14.0	15.0	16.1	17.5	19.0	20.7	22.8
11months	13.9	14.9	16.0	17.3	18.8	20.5	22.6
1year	13.8	14.8	15.9	17.1	18.6	20.3	22.3
1year and 1month	13.7	14.7	15.7	17.0	18.4	20.1	22.1
1year and 2months	13.6	14.6	15.6	16.8	18.3	19.9	21.9
1year and 3months	13.5	14.5	15.5	16.7	18.1	19.7	21.7
1year and 4months	13.5	14.4	15.4	16.6	18.0	19.6	21.5
1year and 5months	13.4	14.3	15.3	16.5	17.8	19.4	21.4
1year and 6months	13.3	14.2	15.2	16.4	17.7	19.3	21.2
1year and 7months	13.3	14.1	15.1	16.3	17.6	19.2	21.1
1year and 8months	13.2	14.1	15.0	16.2	17.5	19.1	21.0
1year and 9months	13.2	14.0	15.0	16.1	17.4	19.0	20.9
1year and 10months	13.1	13.9	14.9	16.0	17.3	18.9	20.8
1year and 11months	13.0	13.9	14.8	15.9	17.2	18.8	20.7
2years	13.2	14.0	15.0	16.1	17.4	19.0	20.9
2years and 3months	13.1	13.9	14.8	15.9	17.2	18.8	20.7
2years and 6months	12.9	13.7	14.7	15.8	17.0	18.6	20.4
2years and 9months	12.9	13.6	14.6	15.6	16.9	18.4	20.3
3years	12.8	13.6	14.5	15.5	16.8	18.3	20.1
3years and 3months	12.7	13.5	14.4	15.4	16.7	18.2	20.1
3years and 6months	12.7	13.4	14.3	15.4	16.6	18.1	20.0
3years and 9months	12.6	13.4	14.3	15.3	16.6	18.1	20.0
4years	12.6	13.3	14.2	15.3	16.6	18.1	20.1
4years and 3months	12.5	13.3	14.2	15.3	16.6	18.2	20.2
4years and 6months	12.5	13.3	14.2	15.3	16.6	18.2	20.3
4years and 9months	12.4	13.2	14.1	15.3	16.6	18.3	20.5
5years	12.4	13.2	14.1	15.3	16.7	18.4	20.7
5years and 3months	12.3	13.1	14.1	15.3	16.7	18.6	21.0
5years and 6months	12.3	13.1	14.1	15.3	16.8	18.7	21.4
5years and 9months	12.2	13.1	14.1	15.3	16.9	18.9	21.7
6years	12.2	13.1	14.1	15.4	17.0	19.1	22.1
6years and 3months	12.2	13.0	14.1	15.4	17.1	19.3	22.5
6years and 6months	12.1	13.0	14.1	15.4	17.2	19.5	22.9
6years and 9months	12.1	13.0	14.1	15.4	17.2	19.7	23.3

Table B.10 Standard deviation values of BMI-for-age for girls under 7 years of age

In kilograms per square metre

age	-3 *SD*	-2 *SD*	-1 *SD*	median	+1 *SD*	+2 *SD*	+3 *SD*
0 month	10.0	10.9	12.0	13.1	14.2	15.4	16.7
1 months	11.7	12.6	13.5	14.7	15.9	17.3	18.9
2 months	12.7	13.7	14.8	16.1	17.5	19.1	20.9
3 months	13.2	14.3	15.4	16.7	18.3	20.0	22.0
4 months	13.5	14.6	15.7	17.1	18.6	20.5	22.6
5 months	13.7	14.7	15.9	17.3	18.8	20.7	22.9
6 months	13.7	14.8	15.9	17.3	18.9	20.8	23.0
7 months	13.7	14.8	15.9	17.3	18.9	20.7	22.9
8 months	13.7	14.7	15.9	17.2	18.8	20.6	22.8
9 months	13.7	14.6	15.8	17.1	18.6	20.4	22.5
10 months	13.6	14.6	15.7	17.0	18.4	20.2	22.3
11 months	13.5	14.5	15.6	16.8	18.3	20.0	22.0
1years	13.4	14.4	15.4	16.7	18.1	19.8	21.8
1year and 1month	13.4	14.3	15.3	16.5	17.9	19.6	21.6
1year and 2months	13.3	14.2	15.2	16.4	17.8	19.4	21.3
1year and 3months	13.2	14.1	15.1	16.3	17.6	19.2	21.1
1year and 4months	13.1	14.0	15.0	16.2	17.5	19.1	21.0
1year and 5months	13.1	13.9	14.9	16.0	17.4	18.9	20.8
1year and 6months	13.0	13.8	14.8	15.9	17.3	18.8	20.7
1year and 7months	12.9	13.8	14.7	15.9	17.2	18.7	20.5
1year and 8months	12.9	13.7	14.7	15.8	17.1	18.6	20.4
1year and 9months	12.8	13.7	14.6	15.7	17.0	18.5	20.3
1year and 10months	12.8	13.6	14.5	15.6	16.9	18.4	20.2
1year and 11months	12.7	13.5	14.5	15.6	16.8	18.3	20.1
2years	12.9	13.7	14.7	15.8	17.0	18.6	20.4
2years and 3months	12.8	13.6	14.5	15.6	16.9	18.4	20.2
2years and 6months	12.6	13.5	14.4	15.5	16.7	18.2	20.1
2years and 9months	12.6	13.4	14.3	15.4	16.6	18.1	20.0
3years	12.5	13.3	14.2	15.3	16.5	18.1	19.9
3years and 3months	12.4	13.2	14.1	15.2	16.5	18.0	19.9
3years and 6months	12.4	13.2	14.1	15.2	16.5	18.0	19.9
3years and 9months	12.3	13.1	14.0	15.1	16.4	18.0	20.0
4years	12.2	13.0	14.0	15.1	16.4	18.0	20.0
4years and 3months	12.2	13.0	13.9	15.0	16.4	18.0	20.1

Table B.10 continued

In kilograms per square metre

age	-3 *SD*	-2 *SD*	-1 *SD*	median	+1 *SD*	+2 *SD*	+3 *SD*
4years and 6months	12.1	12.9	13.9	15.0	16.4	18.1	20.2
4years and 9months	12.1	12.9	13.9	15.0	16.4	18.1	20.3
5years	12.0	12.9	13.8	15.0	16.4	18.2	20.5
5years and 3months	12.0	12.8	13.8	15.0	16.4	18.3	20.6
5years and 6months	11.9	12.8	13.8	15.0	16.5	18.4	20.8
5years and 9months	11.9	12.8	13.8	15.0	16.5	18.4	21.0
6years	11.9	12.7	13.8	15.0	16.6	18.5	21.2
6years and 3months	11.9	12.7	13.8	15.0	16.6	18.6	21.4
6years and 6months	11.8	12.7	13.8	15.0	16.6	18.7	21.5
6years and 9months	11.8	12.7	13.8	15.0	16.7	18.8	21.7
